# Proximal ligation of the pulmonary vein stump to prevent postoperative cerebral infarction after a lobectomy^†^

**DOI:** 10.1093/ejcts/ezaf041

**Published:** 2025-02-14

**Authors:** Tomoki Nishimura, Satoru Okada, Kenji Kameyama, Kazuki Honda, Chiaki Nakazono, Ryosuke Tokuda, Shunta Ishihara, Satoshi Teramukai, Masayoshi Inoue, Yasuo Ueshima

**Affiliations:** Department of Thoracic Surgery, Japanese Red Cross Kyoto Daiichi Hospital, Kyoto, Japan; Division of Thoracic Surgery, Department of Surgery, Kyoto Prefectural University of Medicine, Kyoto, Japan; Division of Thoracic Surgery, Department of Surgery, Kyoto Prefectural University of Medicine, Kyoto, Japan; Department of Thoracic Surgery, Japanese Red Cross Kyoto Daiichi Hospital, Kyoto, Japan; Division of Thoracic Surgery, Department of Surgery, Kyoto Prefectural University of Medicine, Kyoto, Japan; Department of Thoracic Surgery, Japanese Red Cross Kyoto Daiichi Hospital, Kyoto, Japan; Department of Thoracic Surgery, Japanese Red Cross Kyoto Daiichi Hospital, Kyoto, Japan; Division of Thoracic Surgery, Department of Surgery, Kyoto Prefectural University of Medicine, Kyoto, Japan; Department of Thoracic Surgery, Japanese Red Cross Kyoto Daiichi Hospital, Kyoto, Japan; Department of Thoracic Surgery, Fukuchiyama City Hospital, Kyoto, Japan; Department of Thoracic Surgery, Japanese Red Cross Kyoto Daiichi Hospital, Kyoto, Japan; Department of Biostatistics, Kyoto Prefectural University of Medicine, Kyoto, Japan; Division of Thoracic Surgery, Department of Surgery, Kyoto Prefectural University of Medicine, Kyoto, Japan; Department of Thoracic Surgery, Japanese Red Cross Kyoto Daiichi Hospital, Kyoto, Japan

**Keywords:** Postoperative cerebral infarction, Lobectomy, Ligation, Pulmonary vein

## Abstract

**OBJECTIVES:**

Our goal was to assess whether proximal ligation of the pulmonary vein stump (PVS) outside the pericardium reduces the incidence of postoperative cerebral infarction (PCI) after a lobectomy.

**METHODS:**

A single-institution retrospective review was conducted of 683 patients undergoing a lobectomy for lung cancer, metastatic lung tumours and benign diseases. Associations among PCI within 90 days, ligation of the PVS and clinicopathological features were analysed. Effects of PVS ligation on PCI were assessed using multivariable regression. PVS length measured on three-dimensional computed tomographic angiography was compared between the non-ligation and ligation groups.

**RESULTS:**

The non-ligation and ligation groups included 356 (January 2010–March 2018) and 327 patients (April 2018–November 2023), respectively. Seven non-ligation group patients (1.96%) had PCIs [3 in a left upper lobectomy (LUL) and left lower lobectomy (LLL) and 1 in a right lower lobectomy]; no ligation group patients suffered PCIs (*P* = 0.016). Univariable analysis showed that LUL, LLL, chronic kidney disease (CKD), heart failure, cardiovascular disease and PVS ligation were significantly associated with PCIs. Multivariable analysis demonstrated that PVS ligation (*P* = 0.034) was correlated with decreased PCI and LUL (*P* = 0.011), LLL (*P* = 0.010) and CKD (*P* = 0.004) with increased PCIs. After ligation, PVS length was shortened in each lobe in a subset analysis of 109 patients.

**CONCLUSIONS:**

Proximal ligation of the PVS outside the pericardium may be an option to prevent a PCI during a lobectomy. Patients with CKD and those undergoing left-sided lobectomy are at higher risk for PCI and may benefit from more intensive management.

## Graphical Abstract

**Figure:**
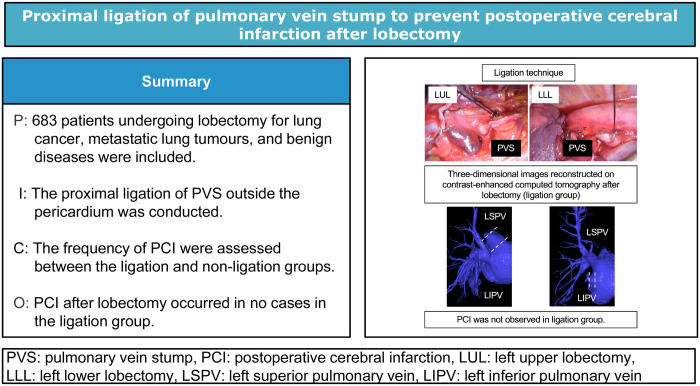

## INTRODUCTION

Postoperative cerebral infarction (PCI) is a complication following lung resection. PCI frequency is 0.27% in Japan [1] and 1.5% in China [2] after lung resection, 10.6 per person-year in Denmark after lobectomy and pneumonectomy [3] and 0.6% in the United States after lobectomy or segmentectomy [4]. Although the frequency of PCI is low, paralysis and other neurological damage can occur, making it difficult for patients to return to daily life. PCI is a potentially life-threatening complication [5, 6]; therefore, perioperative management requires further investigation. Thromboembolic mechanisms involving thrombus in the pulmonary vein stump (PVS) and left atrium are thought to contribute to PCI after a lobectomy [7, 8]. Thrombus formation, associated with a long PVS and blood flow congestion [7, 9], may be reduced through surgical interventions [10, 11].

Although surgical intervention can shorten the PVS and may prevent PCI [12, 13], no standard intervention has been established. The reported frequency of PCI after a left upper lobectomy (LUL) (0.39–4.2%) is higher than that after a left lower lobectomy (LLL) (0.34–0.7%) [1, 6, 14]. PCI has also been reported after a right-sided lobectomy [1, 14]. Therefore, the optimal type of lobectomy for prevention remains debatable. Hypothesizing that proximal ligation could reduce PVS length and decrease thrombus formation and PCI incidences, as of April 2018, we applied proximal ligation of the PVS outside the pericardium for a lobectomy. Accordingly, we examined whether this method was effective in preventing PCI within 90 postoperative days (PODs).

### Patients and methods

This retrospective study included 683 consecutive patients with lung cancer, metastatic lung tumours and benign tumours who underwent a lobectomy at the Japan Red Cross Kyoto Daiichi Hospital between January 2010 and November 2023. Patient characteristics, surgical procedures, postoperative outcomes and proximal ligation of the PVS from databases and medical records were analysed, and PCI occurrence was evaluated. Follow-up was conducted from January 2010 to May 2024. Postoperative complications were assessed using the Clavien–Dindo classification [15]. Complications of grade ≥II were included in the present study. A neurologist diagnosed PCI as an embolic stroke of undetermined source. The main outcome data were collected through manual review of patient records.

### Surgical techniques for pulmonary vein division and ligation

**Figure 1:**
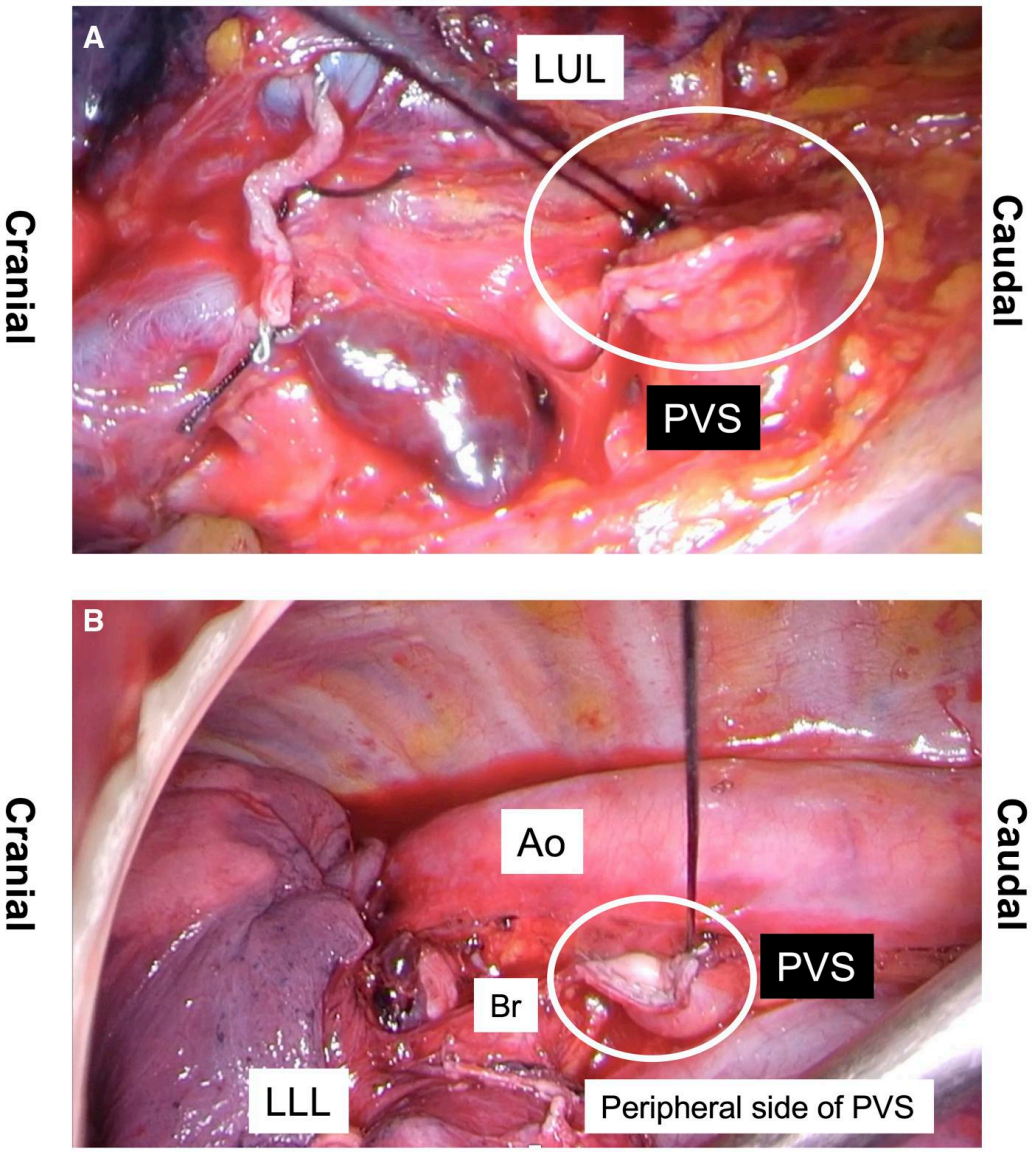
Pulmonary vein stump ligation technique. The pulmonary vein was ligated (**A**) after stapling the pulmonary vein in a left upper lobectomy and (**B**) before stapling the pulmonary vein in other lobectomies. A left lower lobectomy is shown. Ao: aorta; Br: bronchus; LLL: left lower lobectomy; LUL: left upper lobectomy; PVS: pulmonary vein stump.

Lobectomies were performed under complete video-assisted thoracic surgery. In cases of locally advanced lung cancer, uncontrolled bleeding or severe pleural adhesions, open thoracotomy was performed. In patients treated between April 2018 and November 2023 (ligation group), proximal ligation of the pulmonary vein (PV) outside the pericardium was performed using a 2–0 silk thread. In the LUL, the PV was dissected with an endostapler before the PVS was clamped using forceps and ligated with a 2–0 silk thread. In other lobectomies, proximal ligation of the PV outside the pericardium was performed with a 2–0 silk thread before the vein was dissected using an endostapler. In LUL and LLL, the proximal side of the PVS was exposed to the pericardial reflection. The peripheral side of the PV was cut or divided using an endostapler (Fig. 1, Videos 1 and 2). All operations were supervised by a senior thoracic surgeon (Y.U.).

### Pulmonary vein stump length analysis

**Figure 2:**
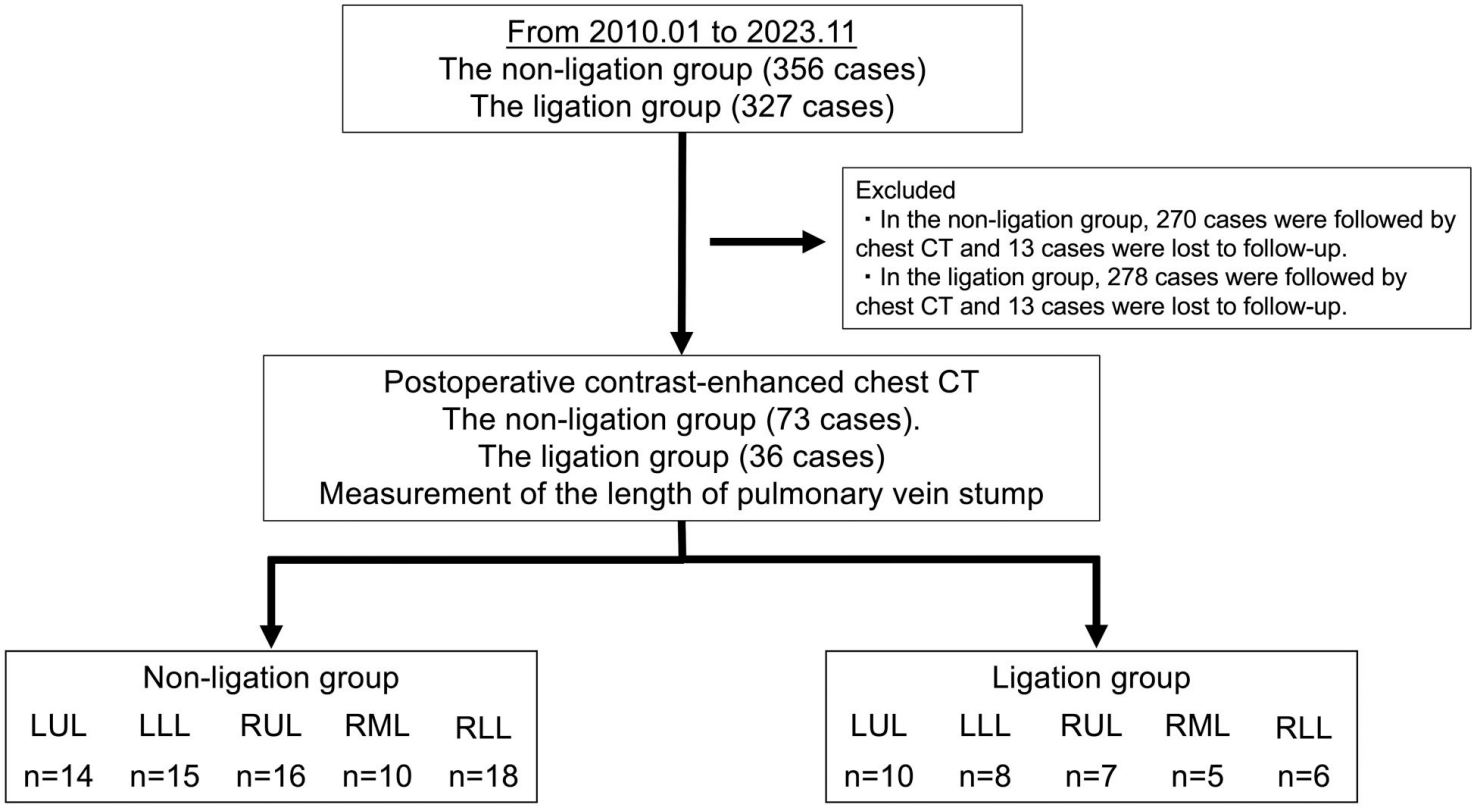
The flow chart of patient selection for pulmonary vein stump length analysis. CT: computed tomography; LLL: left lower lobectomy; LUL: left upper lobectomy; RLL: right lower lobectomy; RML: right middle lobectomy; RUL: right upper lobectomy.

**Figure 3:**
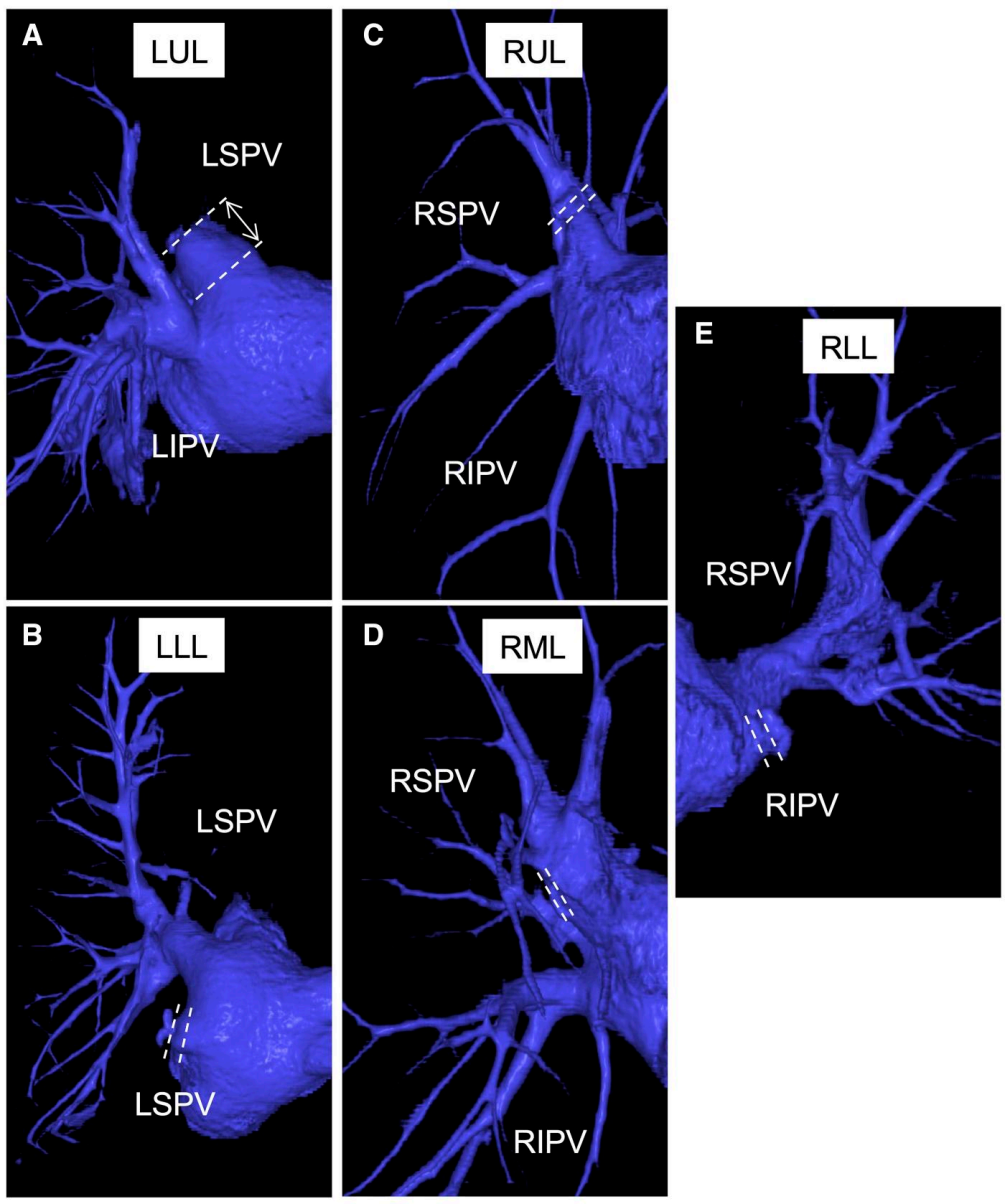
Three-dimensional images reconstructed on contrast-enhanced computed tomography after a lobectomy. Pulmonary vein stump length was measured between the white dotted lines. (**A**) Left upper lobectomy with pulmonary vein stump ligation. (**B**) Left lower lobectomy with pulmonary vein stump ligation. (**C**) Right upper lobectomy without pulmonary vein stump ligation. (**D**) Right middle lobectomy without pulmonary vein stump ligation. (**E**) Right lower lobectomy without pulmonary vein stump ligation. LIPV: left inferior pulmonary vein; LLL: left lower lobectomy; LSPV: left superior pulmonary vein; LUL: left upper lobectomy; RIPV: right inferior pulmonary vein; RLL: right lower lobectomy; RSPV: right superior pulmonary vein; RUL: right upper lobectomy.

The length of the PVS was measured in a subset analysis of 109 of the 683 patients after a lobectomy. Three-dimensional computed tomographic angiography was reconstructed from contrast-enhanced computed tomography (CT) images (Synaps Vincent ver.6.7; FujiFilm, Tokyo, Japan) after a lobectomy in patients in the non-ligation and ligation groups who underwent contrast-enhanced CT (32-slice CT scanner; Canon Medical System, Otawara, Japan) by the sixth postoperative month. The PVS length was measured from the stump to the left atrium in the LUL, LLL and right lower lobectomy (RLL). Right superior PV lengths after a right upper lobectomy and a right middle lobectomy were measured from the stump to the junction with the remaining branches. In the ligation group, the distance from the ligation site to the left atrium or the bifurcation of the right superior PV was measured (Fig. 2A–E). The view that showed the longest PVS was used. The scheme of PVS length analysis is shown in Fig. 3.

### Statistical analyses

Among patient characteristics between the non-ligation and ligation groups, continuous variables were compared using an unpaired *t*-test, and nominal variables were compared using the χ^2^ test or the Fisher exact test when the value of any cell was <5. In the comparison of patient characteristics between non-PCI and PCI groups, an unpaired *t*-test for continuous variables and the Fisher exact test for nominal variables were used. Continuous variables were dichotomized using median values and used as binary variables. Univariable analyses were performed to investigate whether patient characteristics or surgical factors were associated with PCI. Odds ratios and confidence intervals were estimated using a logistic regression analysis. *P* < 0.05 indicated statistical significance. Multivariable analysis was performed with modifications using the Firth method [16]. Variables with *P* < 0.1 in the univariable analyses were entered into the logistic regression model to identify factors independently associated with PCI. Statistical analyses were performed using EZR software [17]. The Student *t*-test was used to analyse PVS length.

### Ethics statement

This study was approved by the ethics committee of the Japanese Red Cross Kyoto Daiichi Hospital, Kyoto, Japan (IRB no. 1550). The requirement for informed consent was waived due to the retrospective nature of the study.

## RESULTS

### Patient characteristics

Table 1 presents the characteristics of all 683 patients. The non-ligation group comprised 356 patients, and proximal ligation of the PVS was performed in 327. PCI occurred in 7 patients (1.9%) in the non-ligation group and in no patients in the ligation group (*P* = 0.016). The median onset of PCI was 1 (0–15) days, and all cases occurred within 30 PODs. One of the 7 patients had a history of cerebral infarction. Carotid artery stenosis was not detected in any patient, and no thrombus was detected in the left atrium on transthoracic echocardiography. Contrast-enhanced CT was performed after the onset of PCI in 2 cases, and no thrombus at the PVS was found in either case. Six patients with hemiplegia, 2 with aphasia and 1 with total blindness were observed. The treatment for PCI was 5 cases of emergency neuroendovascular revascularization and 1 case of anticoagulant and antiplatelet therapy (Supplementary Material, Table S1). The operative duration and amount of blood loss were significantly higher in the non-ligation group, whereas age, body mass index, smoking status and history of hypertension were significantly higher in the ligation group. No significant differences were found between the non-ligation and ligation groups regarding sex, performance status, carcinoembryonic antigen, other past medical history, oral anticoagulants or antiplatelet drugs, neoadjuvant chemotherapy, approach, procedure, pathological status or postoperative complications.

**Table 1:** Patient characteristics

Variable	Non-ligation (*n* = 356)	Ligation (*n* = 327)	*P*-value
Age (years)	71.0 (65–76)	72 (67–77)	<0.001^a^
Sex (male)	213 (59.8)	202 (61.8)	0.63^b^
Performance status (0)	296 (83.1)	266 (81.3)	0.59^b^
Body mass index (kg/m^2^)	21.8 (19.9–24.2)	22.5 (20.3–24.7)	0.013^a^
Smoking (pack years)	25 (0–50)	30.5 (0–51.5)	0.045^a^
Carcinoembryonic antigen (ng/ml)	3.8 (2.5–6.4)	3.4 (2.0–5.9)	0.024^a^
Hypertension (yes)	156 (43.8)	166 (50.8)	0.078^b^
Diabetes mellitus (yes)	68 (19.1)	71 (21.7)	0.44^b^
Hyperlipidaemia (yes)	85 (23.9)	115 (35.2)	0.001^b^
Chronic kidney disease (stages 3–5) (yes)	58 (16.3)	52 (15.9)	0.91^b^
Cardiovascular disease (yes)	56 (15.7)	63 (19.3)	0.22^b^
Preoperative AF (yes)	17 (4.8)	13 (4.0)	0.71^b^
Heart failure (yes)	16 (4.5)	11 (3.4)	0.55^b^
Preoperative cerebral infarction (yes)	18 (5.1)	25 (7.6)	0.20^b^
Anticoagulant (yes)	16 (4.5)	17 (5.2)	0.72^b^
Antiplatelet agent (yes)	47 (13.2)	52 (15.9)	0.32^b^
Neoadjuvant chemotherapy (yes)	14 (3.9)	9 (2.8)	0.52^b^
Postoperative cerebral infarction (yes)	7 (1.9)	0 (0)	0.016^c^
Left upper lobectomy	57 (16.0)	48 (14.6)	0.58^b^
Left lower lobectomy	55 (15.4)	58 (17.7)	0.66^b^
Right upper lobectomy	130 (36.6)	121 (37.0)	0.23^b^
Right middle lobectomy	31 (8.7)	40 (12.2)	0.059^b^
Right lower lobectomy	83 (23.3)	60 (16.1)	0.051^b^
Approach (open)	49 (13.8)	37 (18.4)	0.35^b^
Operative duration (min)	306.5 (258–358.2)	272 (225–329)	<0.001^a^
Blood loss (g)	100 (25–230)	50 (3–115)	<0.001^a^
Histological analysis			0.59^b^
Adenocarcinoma	227 (63.8)	199 (60.9)	
Non-adenocarcinoma	104 (29.2)	99 (30.3)	
Others (metastatic lung tumour, benign diseases)	25 (7.0)	29 (8.9)	
Metastasis of mediastinal lymph nodes (yes)	24 (6.7)	20 (6.1)	0.65^b^
Postoperative empyema (yes)	11 (3.1)	5 (1.5)	0.21^b^
Postoperative AE-IP (yes)	2 (0.6)	5 (1.5)	0.26^c^
Postoperative pneumonia (yes)	6 (1.7)	11 (3.1)	>0.99^b^
Pleurodesis (yes)	37 (10.4)	38 (11.6)	0.62^a^
Postoperative AF (yes)	20 (5.6)	22 (6.7)	0.63^a^
Chest drainage (day)	2 (2–3)	2 (2–3)	0.76^a^
Reoperation (yes)	8 (2.2)	6 (1.8)	0.79^b^

Values in parentheses indicate a reference in categorical variables or a unit in continuous variables. Categorical data are shown as numbers (%) and continuous data as mean (quartile).

a Student *t*-test.

b χ^2^ test.

c Fisher exact test.

AE-IP: acute exacerbation of interstitial pneumonia; AF: atrial fibrillation.

### Predictors of postoperative cerebral infarction

Univariable analysis was conducted between the PCI (7 cases) and non-PCI groups (676 cases). The incidences of CKD and heart failure were significantly higher in the PCI group, and the incidence of proximal ligation of the PVS was significantly higher in the non-PCI group. No significant differences were found between the 2 groups in age, sex, performance status, body mass index, smoking, carcinoembryonic antigen levels, other past medical history, oral anticoagulants or antiplatelet drugs, neoadjuvant chemotherapy, surgical factors, pathological status or postoperative complications (Table 2). Multivariable analyses assessed the impact of PVS ligation on PCI by adjusting for significant factors identified in univariable analyses (LUL, LLL, CKD, cardiovascular disease and heart failure), which showed that ligation of PVS was associated with a decrease in PCI (odds ratio = 0.070, 95% confidence interval = 0.006–0.81, *P* = 0.033), whereas LUL (*P* = 0.010), LLL (*P* = 0.012) and CKD (*P* = 0.003) were associated with an increase in PCI (Table 3). The frequencies of PCI in patients with and without CKD were 4.5% (5/110) and 0.3% (2/573), respectively.

**Table 2:** Characteristics of the postoperative cerebral infarction and non-postoperative cerebral infarction groups

Variable	PCI (−) (*n* = 676)	PCI (+) (*n* = 7)	*P*-value
Age (years)	71.0 (65–76)	74.0 (73–76)	0.32^a^
Sex (male)	412 (60.9)	3 (42.9)	0.44^b^
Performance status (0)	558 (82.5)	4 (57.1)	0.22^b^
Body mass index (kg/m^2^)	22.2 (20.1–24.5)	20.3 (18.95–22.1)	0.14^a^
Smoking (pack years)	27 (0–51.5)	19.5 (0–52.5)	0.88^a^
Carcinoembryonic antigen (ng/ml)	3.5 (2.3–6.2)	3.2 (1.7–6.1)	0.61^a^
Hypertension (yes)	319 (47.2)	3 (42.9)	>0.99^b^
Diabetes mellitus (yes)	138 (20.4)	1 (14.3)	>0.99^b^
Hyperlipidaemia (yes)	197 (29.1)	3 (42.9)	0.42^b^
Chronic kidney disease (stages 3–5) (yes)	105 (15.5)	5 (71.4)	0.002^b^
Cardiovascular disease (yes)	116(17.2)	3 (42.9)	0.10^b^
Preoperative AF (yes)	29 (4.3)	1 (14.3)	0.27^b^
Heart failure (yes)	25 (3.7)	2 (28.6)	0.028^b^
Preoperative cerebral infarction (yes)	112 (17.5)	3 (42.9)	0.11^b^
Anticoagulant (yes)	33 (4.9)	0 (0)	>0.99^b^
Antiplatelet agent (yes)	97 (14.3)	2 (28.6)	0.26^b^
Neoadjuvant chemotherapy (yes)	23 (3.6)	0 (0)	>0.99^b^
Proximal ligation of PVS	327 (48.4)	0 (0)	0.016^b^
Approach (open)	85 (12.6)	1 (14.3)	>0.99^b^
Left upper lobectomy	102 (15.1)	3 (42.9)	0.077^b^
Left lower lobectomy	110 (16.3)	3 (42.9)	0.093^b^
Operative duration (min)	287 (240–347)	299 (292–332)	0.22^a^
Blood loss (g)	70 (3–200)	50 (26.5–222.5)	0.88^a^
Histological analysis			>0.99^b^
Adenocarcinoma	421 (62.3)	5 (71.4)	
Non-adenocarcinoma	201 (29.7)	2 (28.6)	
Others (metastatic lung tumour, benign diseases)	54 (8.0)	0 (0)	
Metastasis of mediastinal lymph nodes (yes)	44 (6.5)	0 (0)	>0.99^b^
Postoperative empyema (yes)	16 (2.4)	0 (0)	>0.99^b^
Postoperative AE-IP (yes)	7 (1.0)	0 (0)	>0.99^b^
Postoperative pneumonia (yes)	17 (2.5)	0 (0)	>0.99^b^
Pleurodesis (yes)	75 (11.1)	0 (0)	>0.99^b^
Postoperative AF (yes)	42 (6.2)	0 (0)	>0.99^b^
Chest drainage (day)	2 (1–3)	2 (2–2)	0.11^a^
Reoperation (yes)	14 (2.1)	0 (0)	>0.99^b^

Values in parentheses indicates a reference in categorical variables or a unit in continuous variables. Categorical data are shown as numbers (%) and continuous data, as the mean (quartile).

a Student *t*-test.

b Fisher exact test.

AE-IP: acute exacerbation of interstitial pneumonia; AF: atrial fibrillation; PCI: postoperative cerebral infarction; PVS: pulmonary vein stump.

**Table 3:** Multivariable analyses for predictors of postoperative cerebral infarction after lobectomy

Variable		Odds ratio	95% CI	*P*-value^a^
Proximal ligation of PVS		0.063	0.005–0.81	0.034
Chronic kidney disease	Yes	8.96	2.04–39.4	0.004
Heart failure	Yes	5.92	0.92–38.1	0.061
Cardiovascular disease	Yes	1.42	0.30–6.78	0.66
Surgical procedure				
Right-sided lobectomy		Ref		
Left upper lobectomy	Yes	11.8	1.75–79.9	0.011
Left lower lobectomy	Yes	12.1	1.81–81.2	0.010

a *P*-value in multivariable logistic analysis performed with modifications of the Firth method.

CI: confidence interval; PVS: pulmonary vein stump.

### Length of the pulmonary vein stump

As a subset analysis (Table 4), PVS length was measured for patients who underwent contrast-enhanced chest CT within 6 months postoperatively. Ligation shortened PVS length in each lobe. LUL had the longest PVS and the largest reduction in PVS length by ligation.

**Table 4:** Pulmonary vein stump length in the non-ligation and ligation groups

Procedure	*N*	Length of PVS (mm)	*P*-value
LUL			<0.001
Non-ligation	14	20.1 (17.3–23.6)	
Ligation	10	13.2 (10.8–14.5)	
LLL			<0.001
Non-ligation	15	10.1 (9.4–12.0)	
Ligation	8	5.1 (4.8–7.2)	
RUL			0.001
Non-ligation	16	6.4 (5.5–8.8)	
Ligation	7	2.8 (2.2–3.4)	
RML			0.002
Non-ligation	10	4.5 (4.0–5.4)	
Ligation	5	1.9 (1.6–1.9)	
RLL			0.001
Non-ligation	18	6.9 (6.0–8.2)	
Ligation	6	2.8 (1.7–3.8)	

The *P*-value was calculated using an unpaired *t*-test.

LLL: left lower lobectomy; LUL: left upper lobectomy; N:number; PVS: pulmonary vein stump; RLL: right lower lobectomy; RML: right middle lobectomy; RUL: right upper lobectomy.

## DISCUSSION

In this study, proximal ligation of the PVS outside the pericardium was performed to prevent PCI after a lobectomy in each lobe, which shortened PVS length and reduced PCI significantly. Bleeding and cardiac tamponade complications on the proximal side of the PV have been reported in the LUL [18]; this possibility was mitigated in our study because the PV was not transected on the proximal side. In other lobectomies, proximal ligation of the PV outside the pericardium with a 2–0 silk thread was performed before the PV was dissected using an endostapler. Proximal ligation was easily performed during each lobectomy. Operative duration was not prolonged in the ligation group. Therefore, proximal ligation of the PVS outside the pericardium can be performed safely and conveniently. Our multivariable analysis of the non-PCI and PCI groups showed that proximal ligation of the PVS decreased PCI, and an LUL, an LLL and a history of CKD increased PCI.

Previously, PV ligation with an LUL resection reduced thrombus formation frequency in the PVS [10]. Nakano *et al.* [11] performed proximal ligation of the PVS, reducing thrombus formation at the PVS in the LUL. Watanabe *et al.* [19] reported that proximal ligation of the PVS in all lobectomies reduced the frequency of thrombus formation in the PVS after an LUL. However, none of these reports showed a reduction in PCI. Although proximal ligation of the PVS outside the pericardium was reported to prevent PCI within 30 days of an LUL [13], the report did not show that it prevented PCI during each lobectomy. In this study, PCI did not occur in any of the cases with ligation.

Thrombus formation in the PVS is a well-known cause of PCI [5]. Ohtaka *et al.* [7] reported PVS length after lobectomy as 17.1 (11.5–26.0) mm in an LUL, 5.4 (3.9–11.0) mm in an LLL and 5.0 (3.1–9.0) mm in RLL. Miyoshi *et al.* [10] also measured a PVS length of 17.5 (7.1–27.7) mm in an LUL, 5.85 (1.7–12.8) mm in an LLL, 3.65 (0.8–11.0) mm in the RLL, 3.85 (1.4–9.5) mm in the right upper lobectomy and 2.5 (0.8–5.5) mm in the right middle lobectomy. Hattori *et al.* [5, 20] reported that a thrombus at the PVS within 90 PODs was associated with left-sided pulmonary resection, especially an LUL. Our study showed that the LUL in the non-ligation group had the longest PVS, similar to that reported by Ohtaka *et al.* PVS length was shortened in each lobe. Nakano *et al.* [11] reported a PVS length of 19.1 (13.5–27.6) mm in the non-ligation group and 8.5 (5.0–15.3) mm in the ligation outside the pericardial groups in the LUL. PVS length showed a shorter trend than that observed in our study, possibly due to the proximal ligation of the PVS after transection of the left superior PV. A previous report showed that the proximal ligation of the PVS outside the pericardium in the LUL effectively prevented thrombus formation and PCI [13]. Our study showed that shortening the PVS by ligation may reduce PCI caused by thrombus formation in the PVS. Ligation shortened the PVS length. However, because this was a subset analysis and the PVS length after an LUL was still longer than after other lobectomies, we could not conclude that shortening the PVS length is associated with the occurrence of PCI.

Prevention and treatment of PCI, besides proximal ligation of the PVS, include anticoagulant treatment and surgical removal of the thrombus. Nakano *et al.* [21] administered anticoagulant therapy with continuous intravenous heparin infusion to prevent thrombus formation. However, they reported PCI onset on POD 8. Ohira *et al.* [8] surgically removed a thrombus at the PVS, which developed 6 months after the LUL. It is difficult to determine when to discontinue anticoagulants. Thrombus extraction with the patient under general anaesthesia is highly invasive. Preventing thrombus formation in the PVS is crucial, and proximal PVS ligation outside the pericardium may be an option for that.

Cardiovascular disease risk in end-stage renal disease is 5 to 30 times higher than in the general population [22]. Patients with CKD (stages 3–4) are at an increased risk of venous thromboembolism [23]. Although the associations between CKD and venous thromboembolism are unclear, body mass index, C-reactive protein and D-dimer levels have been reported as biomarkers [24]. Although the causal relationship between thrombus formation in the PVS and CKD remains unclear, an association between venous thrombus formation and CKD has been reported. In this study, patients in the ligation group with a history of CKD did not undergo PCI. Thus, patients with CKD, who are at high risk for PCI, may be candidates for PVS ligation.

This study has some limitations. First, it was a retrospective single-centre study with PCI occurring in a small number of patients. A prospective multicentre study is required to further evaluate the relationship between proximal ligation of the PVS and PCI prevention. Second, the main surgeons changed throughout the study period; however, because the same senior surgeon (Y.U.) supervised all operations, the quality was consistent. Third, the non-ligation and ligation group operations were performed in different periods, which might have introduced time-related bias. Although potential biases regarding improvements in surgical equipment also exist, variations were not observed in terms of surgical management, which is also attributable to 1 surgeon supervising all procedures. Anticoagulation and low-dose heparin were not routinely administered for thromboprophylaxis in the perioperative period; there were no policy changes during the observation period. The usefulness of perioperative anticoagulant therapy for preventing venous thrombosis in patients undergoing pulmonary resection has not been verified in Japan [25]. Racial differences in coagulation capacity are well-documented, with Asians, including the Japanese, generally experiencing more bleeding complications than coagulation issues compared to Western populations. Studies have reported a higher incidence of cerebral haemorrhage in Asians receiving anticoagulants [26], with Japanese individuals having a fourfold higher risk than Western populations [27]. Consequently, the Japanese guidelines for the diagnosis, treatment and prevention of pulmonary thromboembolism and deep vein thrombosis prioritize physiotherapy as the primary thromboprophylaxis method [28]. Therefore, perioperative anticoagulants are not routinely administered in Japan. In contrast, the European Society of Thoracic Surgeons and the American Association of Thoracic Surgeons suggested using extended prophylaxis for 28 to 35 days rather than in-hospital prophylaxis only in patients undergoing lobectomy or segmentectomy at moderate or high risk of thrombosis [29]. Guidelines for enhanced recovery postoperatively also recommend 4 weeks of anticoagulation to prevent venous thrombosis after lung resection [30]. Due to these differences, it is not possible to determine whether proximal ligation reduces PVS thrombosis in Western countries where anticoagulants are used more routinely. However, to the best of our knowledge, no reports have indicated that prophylactic anticoagulants prevent PCI. A multicentre randomized phase 2 trial is currently underway to evaluate the efficacy of postoperative heparin in preventing PVS thrombus after left-sided anatomical pulmonary resection [31], which may provide evidence in the future. Fourth, PVS length was not measured in all cases, leading to selection bias, and the timing of contrast-enhanced chest CT was inconsistent and did not consider the effect of PVS shortening in the natural course. Fifth, the multivariable logistic regression analysis showed that CKD was significantly associated with PCI. Analysis of databases with larger numbers of cases would be needed to analyse all potential cofounding biases, including CKD.

The aforementioned limitations notwithstanding, performing proximal ligation of the PVS in all types of lobectomies and assessing PCI incidence represent significant strengths of this study.

## CONCLUSION

PCI was significantly associated with a left-sided lobectomy, a history of CKD and PVS ligation outside the pericardium. PVS ligation may be an option for preventing PCI in a lobectomy. Patients with CKD and those undergoing a left-sided lobectomy are at higher risk for PCI and may benefit from more intensive management. Further studies on PCI prevention, including PVS ligation and anticoagulation therapy, are warranted.

## Supplementary Material

ezaf041_Supplementary_Data

## Data Availability

The data underlying this article are available in the article and supplementary material.

## ABBREVIATIONS

CKD: Chronic kidney disease
CT: Computed tomography
LLL: Left lower lobectomy
LUL: Left upper lobectomy
PCI: Postoperative cerebral infarction
POD: Postoperative days
PV: Pulmonary vein
PVS: Pulmonary vein stump
RLL: Right lower lobectomy
RML: Right middle lobectomy
