# Supplementary material for: Proximal ligation of the pulmonary vein stump to prevent postoperative cerebral infarction after a lobectomy
Source: Eur J Cardiothorac Surg. 2025 Feb 14;67(3):ezaf041. doi: 10.1093/ejcts/ezaf041 (PMC11997803; doi:10.1093/ejcts/ezaf041)
Supplement: ezaf041_Supplementary_Data [file ezaf041_Supplementary_Data.zip › Supplementary_table_S1.docx]

Supplementary Table 1. Characteristics of seven patients with PCI

|  | **Case 1** | **Case 2** | **Case 3** | **Case 4** | **Case 5** | **Case 6** | **Case 7** |
| --- | --- | --- | --- | --- | --- | --- | --- |
| **Age** | 74 | 79 | 52 | 76 | 74 | 72 | 76 |
| **Sex** | male | male | female | female | female | male | male |
| **Pathological diagnosis** | Ad | Sq | Ad | Sq | Ad | Ad | Ad |
| **History of cerebral infarction** | yes | no | no | no | no | no | no |
| **Preoperative Af** | no | yes | no | no | no | no | no |
| **Procedure** | LUL | LUL | LUL | LLL | LLL | LLL | RLL |
| **Carotid echo** | NA | no thrombus | no thrombus | NA | no thrombus | no thrombus | no thrombus |
| **Contrast-enhanced chest CT** | no | no | yes (POD16) | no | no | no | yes (POD7) |
| **Thrombus in the PVS** | NA | NA | none | NA | NA | NA | none |
| **Other embolic resources** | np | np | np | np | np | np | np |
| **Duration from surgery to onset** | POD 1 | POD 0 | POD 15 | POD 7 | POD 1 | POD 1 | POD 14 |
| **Postoperative Af** | no | no | no | no | no | no | no |
| **Symptoms** | total blindness | hemiplegia | hemiplegia, aphasia | hemiplegia, aphasia | hemiplegia | hemiplegia | hemiplegia |
| **Treatment** | Anti-platelet | Anticoagulant | ENER | ENER | ENER | ENER | ENER |
| **Death due to PCI** | no | no | no | yes | no | no | no |

Ad: adenocarcinoma; Sq: squamous cell carcinoma; LUL: left upper lobectomy; LLL: left lower lobectomy; RLL: right lower lobectomy; CT: computed tomography; POD: postoperative day; PVS: pulmonary vein stump; Af: atrial fibrillation; ENER: emergency neuroendovascular revascularization; and PCI: postoperative cerebral infarction.
