# Supplementary material for: Proximal ligation of the pulmonary vein stump to prevent postoperative cerebral infarction after a lobectomy
Source: Eur J Cardiothorac Surg. 2025 Feb 14;67(3):ezaf041. doi: 10.1093/ejcts/ezaf041 (PMC11997803; doi:10.1093/ejcts/ezaf041)
Supplement: ezaf041_Supplementary_Data [file ezaf041_Supplementary_Data.zip › Supplementary_table_S3.docx]

Supplementary Table 3. Patient characteristics after IPTW

| **Variable** | **Non-ligation (n=618.3)** | **Ligation (n=632.7)** | **SMD** |
| --- | --- | --- | --- |
| Age (years) | 70.1 (65–76) | 69.9 (65–76) | 0.013 |
| Sex (male) | 377.2 (61.0) | 394.8 (62.4) | 0.024 |
| Performance status (0, 1) | 512.6 (82.9) | 520.1 (82.2) | 0.004 |
| Body mass index (kg/㎡) | 22.5 (20.3–24.7) | 22.5 (20.1–24.3) | 0.004 |
| Smoking (pack years) | 33 (0–55) | 33.7 (0–50) | 0.016 |
| Carcinoembryonic antigen (ng/mL) | 6.5 (2.1–6.2) | 6.4 (2.6–6.4) | 0.004 |
| Hypertension (yes) | 269.6 (43.6) | 323.9 (51.2) | 0.019 |
| Diabetes mellitus (yes) | 118.7 (19.2) | 137.9 (21.8) | 0.019 |
| Hyperlipidaemia (yes) | 147.2 (23.8) | 219.5 (34.7) | 0.015 |
| Chronic kidney disease (Stage 3-5) (yes) | 103.9 (16.8) | 94.3 (14.9) | 0.009 |
| Cardiovascular disease (yes) | 100.2 (16.2) | 94.3 (14.9) | 0.009 |
| Preoperative Af (yes) | 30.3 (4.9) | 27.2 (4.3) | 0.020 |
| Heart failure (yes) | 28.4 (4.6) | 20.9 (3.3) | 0.009 |
| Preoperative cerebral infarction (yes) | 32.2 (5.2) | 50.0 (7.9) | 0.014 |
| Anticoagulant (yes) | 24.7 (4.0) | 35.4 (5.6) | 0.032 |
| Antiplatelet agent (yes) | 75.4 (12.2) | 106.3 (16.8) | 0.013 |
| Neoadjuvant chemotherapy (yes) | 24.7 (4.0) | 19.0 (3.0) | 0.032 |
| Left upper lobectomy | 98.3 (15.9) | 100.0 (15.8) | <0.001 |
| Left lower lobectomy | 96.5 (15.6) | 104.4 (16.5) | 0.003 |
| Right upper lobectomy | 226.3 (36.6) | 231.6 (36.6) | 0.022 |
| Right middle lobectomy | 52.6 (8.5) | 70.9 (11.2) | 0.002 |
| Right lower lobectomy | 145.3 (23.5) | 125.3 (19.8) | 0.027 |
| Approach (open) | 81.0 (13.1) | 62.6 (9.9) | 0.004 |
| Operative duration (min) | 302.3 (252–348) | 305.3 (239–352) | 0.025 |
| Blood loss (g) | 168.2 (3–200) | 168.2 (3–150) | <0.001 |
| Histology  adenocarcinoma  non-adenocarcinoma  others (metastatic lung tumour, benign diseases) | 589.9 (95.4)  26.6 (4.3)  1.8 (0.3) | 586.7 (92.7)  29.4 (4.6)  16.6 (2.6) | 0.019 |
| Metastasis of mediastinal lymph nodes (yes) | 43.3 (7.0) | 41.8 (6.6) | 0.012 |
| Postoperative empyema (yes) | 16.7 (2.7) | 8.2 (1.3) | 0.005 |
| Postoperative IP-AE (yes) | 3.7 (0.6) | 10.8 (1.7) | 0.006 |
| Postoperative pneumonia (yes) | 9.3 (1.5) | 16.5 (2.6) | 0.009 |
| Pleurodesis (yes) | 62.4 (10.1) | 75.9 (11.9) | 0.003 |
| Postoperative Af (yes) | 34.0 (5.5) | 43.7 (6.9) | 0.023 |
| Chest drainage (day) | 3.3 (2–3) | 3.3 (2–3) | 0.021 |
| Reoperation (yes) | 14.8 (2.4) | 10.8 (1.7) | 0.026 |

Values in parentheses indicate a reference in categorical variables or a unit in continuous variables. Categorical data are shown as numbers (%) and continuous data as mean (quartile).

IPTW: inverse probability of treatment weighting; SMD: standardized mean difference; Af: atrial fibrillation; and AE-IP: acute exacerbation of interstitial pneumonia
