# Supplementary material for: Proximal ligation of the pulmonary vein stump to prevent postoperative cerebral infarction after a lobectomy
Source: Eur J Cardiothorac Surg. 2025 Feb 14;67(3):ezaf041. doi: 10.1093/ejcts/ezaf041 (PMC11997803; doi:10.1093/ejcts/ezaf041)
Supplement: ezaf041_Supplementary_Data [file ezaf041_Supplementary_Data.zip › Supplementary_table_S2.docx]

Supplementary Table 2. Patient characteristics before IPTW

| **Variable** | **Non-ligation (n=356)** | | **Ligation (n=327)** | **SMD** |
| --- | --- | --- | --- | --- |
| Age (years) | | 71.0 (65–76) | 72 (67–77) | 0.31 |
| Sex (male) | | 213 (59.8) | 202 (61.8) | 0.029 |
| Performance status (0, 1) | | 350 (98.3) | 317 (96.9) | 0.075 |
| Body mass index (kg/㎡) | | 21.8 (19.9–24.2) | 22.5 (20.3–24.7) | 0.21 |
| Smoking (pack years) | | 25 (0–50) | 30.5 (0–51.5) | 0.19 |
| Carcinoembryonic antigen (ng/mL) | | 3.8 (2.5–6.4) | 3.4 (2.0–5.9) | 0.017 |
| Hypertension (yes) | | 156 (43.8) | 166 (50.8) | 0.15 |
| Diabetes mellitus (yes) | | 68 (19.1) | 71 (21.7) | 0.064 |
| Hyperlipidaemia (yes) | | 85 (23.9) | 115 (35.2) | 0.24 |
| Chronic kidney disease (Stage 3-5) (yes) | | 58 (16.3) | 52 (15.9) | 0.052 |
| Cardiovascular disease (yes) | | 56 (15.7) | 63 (19.3) | 0.14 |
| Preoperative Af (yes) | | 17 (4.8) | 13 (4.0) | 0.028 |
| Heart failure (yes) | | 16 (4.5) | 11 (3.4) | 0.065 |
| Preoperative cerebral infarction (yes) | | 18 (5.1) | 25 (7.6) | 0.11 |
| Anticoagulant (yes) | | 16 (4.5) | 17 (5.2) | 0.077 |
| Antiplatelet agent (yes) | | 47 (13.2) | 52 (15.9) | 0.13 |
| Neoadjuvant chemotherapy (yes) | | 14 (3.9) | 9 (2.8) | 0.054 |
| Left upper lobectomy | | 57 (16.0) | 48 (14.6) | >0.001 |
| Left lower lobectomy | | 55 (15.4) | 58 (17.7) | 0.026 |
| Right upper lobectomy | | 130 (36.6) | 121 (37.0) | 0.001 |
| Right middle lobectomy | | 31 (8.7) | 40 (12.2) | 0.090 |
| Right lower lobectomy | | 83 (23.3) | 60 (16.1) | 0.089 |
| Approach (open) | | 49 (13.8) | 37 (18.4) | 0.10 |
| Operative duration (min) | | 306.5 (258–358.2) | 272 (225–329) | 0.42 |
| Blood loss (g) | | 100 (25–230) | 50 (3–115) | 0.23 |
| Histology  adenocarcinoma  non-adenocarcinoma  others (metastatic lung tumour, benign diseases) | | 227 (63.8)  104 (29.2)  25 (7.0) | 199 (60.9)  99 (30.3)  29 (8.9) | 0.11 |
| Metastasis of mediastinal lymph nodes (yes) | | 24 (6.7) | 20 (6.1) | 0.029 |
| Postoperative empyema (yes) | | 11 (3.1) | 5 (1.5) | 0.10 |
| Postoperative IP-AE (yes) | | 2 (0.6) | 5 (1.5) | 0.099 |
| Postoperative pneumonia (yes) | | 6 (1.7) | 11 (3.1) | 0.078 |
| Pleurodesis (yes) | | 37 (10.4) | 38 (11.6) | 0.058 |
| Postoperative Af (yes) | | 20 (5.6) | 22 (6.7) | 0.060 |
| Chest drainage (day) | | 2 (2–3) | 2 (2–3) | 0.001 |
| Reoperation (yes) | | 8 (2.2) | 6 (1.8) | 0.056 |

Values in parentheses indicate a reference in categorical variables or a unit in continuous variables. Categorical data are shown as numbers (%) and continuous data as mean (quartile).

IPTW: inverse probability of treatment weighting; SMD: standardized mean difference; Af: atrial fibrillation; and AE-IP: acute exacerbation of interstitial pneumonia
